# ‘Practice what you preach’. Perspectives on the involvement of people with dementia and carers in community-based dementia friendly initiatives, a qualitative study

**DOI:** 10.3389/fpsyt.2024.1387536

**Published:** 2024-05-16

**Authors:** Marjolein Thijssen, Linda Dauwerse, Frans Lemmers, Maria Nijhuis-van der Sanden, Ramon Daniels, Maud Graff, Wietske Kuijer-Siebelink

**Affiliations:** ^1^ Radboudumc Research Institute, Scientific Center for Quality of Health (IQ health), Radboud University Medical Center, Nijmegen, Netherlands; ^2^ Radboud Alzheimer Center, Radboud University Medical Center, Nijmegen, Netherlands; ^3^ Department Occupational Therapy, School of Allied Health, HAN University of Applied Sciences, Nijmegen, Netherlands; ^4^ Department of Primary and Community Care, Medical Centre, Radboud University Medical Center, Nijmegen, Netherlands; ^5^ Mentality, Nijmegen, Netherlands; ^6^ Research Centre Assistive Technology in Care, Zuyd University of Applied Sciences, Heerlen, Netherlands; ^7^ School of Education, HAN University of Applied Sciences, Nijmegen, Netherlands; ^8^ Radboudumc Health Academy, Research on Learning and Education, Radboud University Medical Center, Nijmegen, Netherlands

**Keywords:** patient and public involvement, inclusion, dementia-friendly, co-design, assets-based, relational expertise, relational agency, relations

## Abstract

**Introduction:**

People with dementia and their carers experience social stigma and often refrain from social participation. Significant improvement might be achieved by creating Dementia Friendly communities (DFCs) for which dementia friendly initiatives (DFIs) are needed. DFIs are developed by a variation of stakeholders. However, people with dementia and their carers are often unrepresented herein. This study aims to get insight into the perspectives of stakeholders (e.g., health- and social care professionals, volunteers, people with dementia and their carers) about the involvement of people with dementia and their carers during the development and sustainment of DFIs.

**Methods:**

Descriptive qualitative study, using a co-research design with a carer as co-researcher. Nineteen semi-structured interviews with stakeholders, including people with dementia and their carers, were performed. Inductive content analysis took place using Atlas Ti.

**Results:**

Four themes were found: 1) the involvement of people with dementia and their carers is important for both people with dementia and their carers and other stakeholders; 2) personal character traits, life histories, and associated emotions evoke the need for involvement; 3) involvement requires an open, responsive stance and building relationships; and 4) the estimation of one’s own and others’ capacities influences perspectives on involvement. As such, practice what you preach means actively adopting an open, responsive approach and acknowledging the unique abilities and backgrounds of people with dementia and their carers. It emphasizes the importance of actually living by the values you advocate for.

**Conclusion:**

Central to perspectives on involving people with dementia and their carers is the emphasis on working relationally, differing from service-led and pre-structured patient and public involvement (PPI). Working relationally calls for organizational shifts aligned with a rights-based perspective to avoid tokenism, and promotion of user-led organizations with genuine partnerships. Creative methods, problem-solving, and communication skills are essential for the development and sustainment of inclusive, supportive, person-centered DFIs. Future studies should explore the long-term impact of the involvement and working relationally on the well-being of people with dementia and their carers.

## 1 Introduction

Dementia is a progressive cognitive decline, that leads to multifaceted problems especially memory loss, impaired judgment, and difficulty in daily activities. It affects quality of life, strains relationships, and poses challenges for carers. People with dementia may lose independence, and there is no cure, necessitating ongoing support and care (1–3). In recent years there is growing recognition of dementia as an urgent global health issue (4–6), which has led to an increase in dementia friendly communities (DFCs) (7). DFCs aim to include people with dementia and their carers as equal citizens (3, 8, 9). For this, dementia- friendly initiatives (DFI) are initiated as ‘building blocks’ in the advancement of DFCs (10–12). Examples of DFIs include an Alzheimer ‘café’, including people with dementia in existing group activities such as a choir or adapting the public physical environment to people with dementia’s needs. The core of DFIs is that initiatives or activities are adjusted to the specific needs of people with dementia and their carers and other citizens in a local context. Therefore, the development and sustainment of a DFI relies on stakeholder involvement such as local policy officers, healthcare and welfare professionals, volunteers and citizens from the local community (8, 13–15) The input of people with dementia and their carers therein is vital to ensure that a DFI meets their needs; they have local knowledge, can identify barriers and opportunities and bring expertise through experience (3, 8, 16, 17). However, the input of people with dementia and their carers themselves is often limited (13–15, 18–22) due to multiple reasons. First, diminished attention from professionals, policymakers, and researchers, lack of enthusiasm among people with dementia and citizens, and debates about the added values compared to the effort needed, decrease motivation for their input (13, 14, 18, 21). Furthermore, the input and expertise of people with dementia and carers seems to be overshadowed by professional hierarchy, -’hunches’ and -work routines (13, 22). Finally, the input of people with dementia is not easily incorporated and organized into projects’ planning (15, 18–20). It required adaptations which are perceived as too drastic because, for instance, additional meetings are necessary or the project’s pace needs to be adjusted. These barriers were also found in our previous studies within the Mentality Project (see below) (13, 22, 23).

The concept of public and patient involvement (PPI) emphasizes that the perspectives of people with dementia and their carers are crucial, advocating that health and social care research and services should be conducted ‘with’ people, rather than ‘for’ or ‘to’ them (16, 24, 25). PPI, which is still developing, employs various approaches and theoretical frameworks (26, 27)., A well-known model is Arnstein’s typology of citizen participation, which categorizes different ladders of PPI (28). However, this model has been critiqued for its lack of context sensitivity, such as its application in collective processes among all stakeholders (29, 30). Recent research have shown that PPI in dementia involves co-production, learning, and power sharing (22, 27) and needs to be context-sensitive to build relationships (18, 31). The importance of attitudes, feelings, and emotions is highlighted, as PPI requires the careful and inclusive development of relationships where everyone’s contributions are respected and valued (24, 27).

The issue with previous research is that it focused on PPI in dementia research (32–36) and on the implementation of, and barriers to, PPI in DFCs (18–22). Until now, little attention has been paid to studying the underlying perspectives of stakeholders on the involvement of people with dementia and their carers during the development and sustainment of DFIs (e.g. their beliefs, attitude, values and their ideas on involvement of people with dementia and their carers). The same applies for the perspectives of people with dementia and their carers about their own involvement.

With this study, we wanted to address this gap by studying the perspectives of both stakeholders and people with dementia and their carers regarding the involvement of people with dementia and carers during the development and sustainment of DFIs. The central research question of this study was: What are the perspectives of stakeholders, people with dementia and their carers about the involvement of people with dementia and their carers during the development and sustainment of community DFIs? The aim was to both gain insight into the perspectives underlying the previously mentioned barriers and to gain insight into what might support for PPI during the development and sustainment of DFIs.

## 2 Methods

### 2.1 Mentality project

The current study was performed between March 2022 and September 2022 and is the fourth phase of the Mentality Project (i.e., ‘Mentality’; November 2017-October 2022) The aim of Mentality project was to identify the key mechanisms and contextual factors that facilitate development and sustainability of DFIs, thereby enhancing meaningful outcomes for people with dementia and their caregivers within the community (37). The Mentality project was guided by the research team and an advisory panel consisting of experts in the field of dementia and public health, representatives of people with dementia and their caregivers and stakeholders from four Dutch municipalities seeking to become dementia-friendly’.

### 2.2 Approach of this study

This qualitative, exploratory study was based upon a constructivist epistemology and used a critical-emancipatory study design (37–39) with inductive content analysis (40) and a co-research approach (41). A carer with lived experience of caring for someone with dementia (FL) joined the research team, collaborating closely with two academic researchers (MT and LD). Supervision was provided by other team members (RD, RNvdS, MG, and WKS). Our study progressed through five stages that fostered equal partnership and shared decision-making (41–44):

1. Collaboration and Research Planning: The research team, familiar with each other through the advisory panel of Mentality, devised a collaboration plan. The carer’s role was defined based on his interests, skills, and experience, emphasizing the value of a lay perspective. The stage concluded with agreement on the study’s aim, questions, and feasibility.
2. Preparing data collection: MT and LD developed semi-structured interview guides, incorporating content for the interview guide and implications for interview techniques from three preparatory meetings with the co-researcher. He later withdrew from interviewing due to potential emotional conflicts, a previously identified methodological pitfall (42). Three pilot interviews with two professionals and a carer were conducted, audiotaped, and reviewed by MT, LD and FL. Reflection, and feedback on the interview topics and techniques were provided and incorporated in the interview guide. The interview guide was approved by the supervisors.
3. Collecting data: Interviews were held by the researchers MT and LD.
4. Preparing and conducting data analysis: Inductive content analysis took place in four phases; in which the co-researcher contributed a lay perspective (40–42).
5. Disseminating findings: All team members, including the co-researcher, contributed to writing and revising the scientific paper by providing feedback to the first author. The co-researcher also helped disseminate the findings through his network.

The co-researcher received a reward for his contribution and his expenses were reimbursed.

### 2.3 Participants

Eligible participants were either a) health- and social professionals, volunteers, and citizens, who were active in developing and sustaining DFIs or b) people with dementia and their carers who participated in a DFI and/or were involved in the development of a DFI. Study exclusion criteria for all eligible participants were not being able to understand relevant information or communicate verbally.

### 2.4 Recruitment and inclusion of DFIs and participants

Recruitment started with a selection of DFIs, who were invited via the DFCs that previously participated in Mentality. One DFI was recruited via the network of a co-researcher (FL); two DFI’s were recruited by the network of other researchers (LD and MT). In total, three DFIs were included, represented in **Table 1**. The DFIs ‘Meeting center’ and ‘Carers café’ were initiated by professionals, the DFI ‘Table tennis club’ was initiated by volunteers from the club itself. These DFIs were one out of more DFIs that were undertaken in order to advance a city towards a DFC.

**Table 1:** Presentation of participants in the DFIs.

DFI		Interviews			
		Professionals N=	Volunteers N=	Carers N=	People with dementia N=
Meeting center: a social space where people with dementia and their carers can meet others and take part in activities and/or conversations weekly, based on what they are interested in.	Number (Male)	5 (1)	1 (0)	2 (0)	2 (2)
	Age*	A: 4, B:1	A:1	A: 1, B:1	B:1, C:1
	Educational level@	Bsc:4, VET: 1	LVET:1	Bsc:1, VET: 1	Bsc:2
Carers’ café: a social space where carers of people with dementia, can meet other carers and professionals for information, advice, and emotional support once a month.	Number (Male)	2 (1)	2 (2)	1 (0)	1 (1)
	Age*	A: 2	B:2	C: 1	B:1
	Educational level@	U:1, Bsc: 1	Bsc:2	LVET: 1	LVET: 1
Table tennis club: a club for people who like to play table tennis several times per week.	Number (Male)	0	2 (2)	1 (0)	0
	Age*		B:1, C:1	C:1	
	Educational level@		U: 1, Bsc: 1	Bsc: 1	

*Age range: A = 26-64 years, B= 65-74 years, and C= 75-85 years old.

@Educational level: U, university; Bsc, Bachelor of science; VET, Vocational Education and Training; LVET, Lower Vocational Education and Training.

Inclusion proceeded with the recruitment of participants, who were recruited via the key informants for each DFI and then via snowball sampling (45, 46). Thus, key informants were asked to identify other information-rich informants who were active and engaged in the development and implementation of DFIs. Next, participants assisted in the identification of other eligible participants (46). In total, seven professionals were interviewed, including six healthcare- and social professionals and one project leader, and five volunteers, four carers, and three people with dementia were interviewed; all were White, native Dutch people. **Table 1** shows background information of both the DFIs and the participants per DFI.

All participants received verbal and written information and signed an informed consent document before each interview. All participants were interviewed at an appropriate space of their own choice to maintain participant confidentiality (46, 47).

### 2.5 Data collection and analysis

Qualitative data were gathered during individual semi-structured interviews. When preferred by the person with dementia, the carers attended the interview. The participants were initially invited to talk about the importance of ‘their’ DFI and the involvement of people with dementia and carers in this. Concrete examples of PPI supported the interview and were derived from the ‘ladder’ of citizens’ participation (28). This enabled prompting to explore attitudes, experiences, needs, and perceptions regarding PPI in their DFI and how and why these currently (did not) play out in their DFI(s). Other topics address contextual factors, for example, a possible dissonance between formal policies and what happens in practice to capture top-down and bottom-up input on DFIs (26, 48). The interview guide is available in the **Supplementary Material**.

All interviews lasted between 15 and 75 minutes and were recorded and transcribed verbatim. One interview was only 15 minutes because the participants had to leave unexpectedly.

All data were analyzed using an iterative inductive content analysis (40, 49) supported by Atlas Ti (version 23.07), taking place in four phases. In Phase 1, the researchers (MT and LD) and co-researcher (FL) independently read the transcripts of one professional, volunteer, and carer and a person with dementia to become familiar with the data. During an online meeting, they then clarified questions about the data to avoid pitfalls of language barriers and/or misunderstandings (42, 50, 51). In Phase 2, they independently coded these transcripts by underlining relevant pieces of text and writing coding labels/ideas for each on the margins of each interview transcript. The codes could be a word or short phrase that characterized the participant’s opinions, attitudes, experiences, and/or reflections regarding the involvement of people with dementia and/or their carers. Subsequently, the researchers and co- researcher sent each other their transcripts with their annotations. In a second meeting, they discussed the procedure and initial codes to increase the rigor of the coding procedure. The co-researcher was invited to present his interpretation of the data first before it was merged with the interpretations of LD and MT, to make sure that his interpretation was not influenced by the other researchers (41). In Phase 3, the other transcripts were coded and codes were grouped into categories, subthemes and themes by a researcher (MT). In this phase, two meetings were held in which MT disclosed the process of analysis and current findings to LD and FL. During these meetings, they reflected on the analysis and preliminary results in which the co-researcher first gave his reflection independently from LD and MT for the same reason as mentioned in Phase 2. The reflections were incorporated into the ongoing data analysis by MT. Reflections, for examples, pertained to relationships between people with dementia, carers, professionals, and volunteers, and the awareness among the participants how this played out in being or feeling involved, or not. In the final Phase 4, the initial results of six themes were presented to the whole research team. After feedback exchange and critical dialoguing, the initial results were checked against the transcripts and revised into four themes. These were discussed again and recognized as trustworthy by the whole team.

Data collection and analysis was conducted in Dutch.

### 2.6 Reflexivity

The co-researcher (FL) was free to participate in the study. Invited based on his role in Mentality’s advisory group, he was asked if he would like to collaborate with the research team to bring and share his lay perspective from the start to the end of this study. The co-researcher (FL) was the carer of his wife who had frontotemporal dementia. Furthermore, he had 35 years of prison management, addiction care, and probation services experience, and he was currently a volunteer in the field of dementia and international probation educational services. He worked closely with the other executive researchers (e.g., the main researcher, MT, and researcher LD). Researcher LD is experienced in clinical ethics, participation, and empowerment, and in the facilitation of communities of practice to develop and sustain DFIs. She currently works as a supervisor, trainer, and researcher in the field of elderly care. The main researcher (MT) is experienced in client-centred practice and -research in the public and private fields of dementia, and is currently a Ph.D. candidate.

Researchers MT and LD had an understanding of the lived experience of dementia at different stages and were experienced in strategies that support communication with people with dementia (48). They were well-acquainted with the interview guide by conducting pilot interviews and providing and integrating reflection and feedback (see also ‘preparing data collection’ as a step of the co-research (41).

FL, LD and MT were supervised by the other members of the research team (RD, RNvdS, MG, and WKS). Supervision took place during two meetings.

The researchers had five online meetings. During the first meeting, the steps of co-research (41) were discussed, including the co-researcher’s role and his interests, skills and experience, and then incorporated into the research plan. The collaboration between the researchers was launched starting with the input of personal assets and their tacit and explicit expectations about learning (42), such as wanting to give a perspective on the development of a DFC throughout an entire study (FL), embedding research skills combined with experience as a facilitator (LD), and increasing skills in in PPI in research (MT). As such, the steps of co-research were attuned to the co-researcher’s needs and interests. The co-researcher did not follow specific research training, as this did not add to his expectations of learning, and the research team’s focus was on his lay perspective. It was up to the other researchers to communicate effectively, for example, around conducting the interviews and coding the transcripts, rather than make the co-researcher ‘less lay’.

We involved the co‐researcher during all research phases as described in Section 2.1. The main researcher (MT) kept a log with progress of the study and recorded and made notes during meetings with a researcher (LD) and the co-researcher (FL). By using mutual reflective questions and a logbook, the process of a shared decision- making and power balance was monitored in an open evaluation after each meeting by the executive researchers. The records and notes from the meetings were important in the further development of the initial results during data analysis. By an iterative process, they supported reflection on the initial results. Finally, the reflection and the notes were also used in meetings between the main researcher (MT) and supervisors when a co-researcher (FL) and one researcher (LD) were not present, so that their vision was still ‘heard’ and brought in.

## 3 Results

The perspectives of stakeholders, people with dementia, and their carers regarding the involvement of people with dementia and their carers during the development and sustainment of community DFIs were categorized into four themes. Each theme will be described and supported by quotes from the participants. Unless otherwise stated, the results reflect the perspectives of all participants.

### 3.1 Involvement of people with dementia and their carers is important for both people with dementia and their carers and other stakeholders in a DFI

All participants, except one, believed that involvement was important. Knowing the needs of people with dementia and carers was regarded as vital to develop DFIs that aim to support the inclusion of people with dementia and their carers.

People with dementia considered their involvement as self-evident, as a way to express themselves about what is meaningful for them:

*If they are talking about dementia, I want to hear it. I find it very important to talk about it … not only by people who know you and support you but also by people who understand. They are as important as people who know a lot. (person with dementia, DFI 2)*

*I want to know what we are going to do, if we are going for a walk or making candles or something, so I can adjust my clothing. I am used to looking after my clothes to look presentable; that was important in my job. (person with dementia, DFI 1)*

Other stakeholders stated that people with dementia know best what they need:

*And why should people be involved in this? Because they know best what they would be helped with. (volunteer, DFI 2)*

One volunteer did not consider involvement important because she interpreted the active participation of people with dementia in the DFI itself as a confirmation that the DFI was adjusted to their needs:

*SP 1: They always participate. SP 2: They always participate. SP 1: Yes, no one complains or says, ‘I don’t want to do that.’ SP 2: You actually don’t hear any protest at all. SP 1: No. SP 2: Or questions? Objections? Or ‘yes, but…’ SP 1: No, no. It’s good the way it is. (volunteer, DFI 1)*

Besides the primary value for people with dementia, involvement was also considered to be important for other stakeholders. They acknowledged a ‘reciprocal’ reward. From the carers’ perspective, they considered their involvement important to inform the DFI about their relatives’ needs or interests to ensure that they had a ‘good time.’ When carers could trust that their relatives had a good time, they felt better, too:

*Well, to make sure my husband has a good time. Because I’ve heard once that he did not like it. And he is always very much looking forward to it* [the DFI]*. So yes, there is a bit of self-interest. (carer, DFI 1)*

Professionals and volunteers mentioned how the involvement of people with dementia and carers felt rewarding for them because it enabled them to do something that was meaningful for others. That feeling was fulfilling for them and offered enjoyment.

*Lots of fun, insight, yes, also the joy of life*, [feeling] *satisfaction, if you do something that’s really important for the other person. You’re not just messing around. So, it’s really an added value for yourself. (professional, DFI 1)*

However, if involvement felt mandatory and distracted from other activities, this impacted the fulfillment and joy experienced by stakeholders:

*Earlier, it was more spontaneous, and like, ‘yes, we are going to do this and we are going to do that.’ But now it’s feedback in advance, feedback afterwards. And all the spontaneity is gone. SP 1: How is that important to you? SP 2: Well … with spontaneity, there’s no ‘must.’ There is nothing bound to it; it just comes purely from your heart, from your feeling, and that is no longer the case. (volunteer, DFI 1)*

### 3.2 Personal character traits, life histories, and associated emotions evoke the need for involvement

All participants revealed how their perspectives on involvement were shaped by personal traits, experiences, choices, and past and future-expected circumstances. Together with the associated emotions, it created a sense of urgency around wanting to be involved or creating opportunities for others to be involved.

Personal character traits were mentioned by persons with dementia who spoke of personal characteristics and values as a means to be involved:

*SP 1: And do you feel that you can easily say what you like and don’t like here? SP 2: Well, I never mince words at all. SP 1: No? SP 2: No. I think you should keep people informed, and say it honestly. (person with dementia, DFI 1)*

Other participants also mentioned personal characteristics and values that shaped their attitudes and values around involvement:

*The vulnerable … well, that sounds a bit … giving the vulnerable a voice, too. That is, and that is my conviction, and that is also a bit of who I am. (volunteer, DFI 2)*

Personal life experiences with an emotional impact, positive or negative, affected perspectives on the involvement of people with dementia and their carers. One professional talked about how her mother with dementia was treated and how this affected her perspective on involvement during her professional life:

*They asked her* [mother]*: What would you like? She said: ‘That people act normal towards me.’ I saw what this meant for her, being treated like normal, equal. They invited her to have a say, just like everybody else. That was really powerful; my mother was normal. (project leader, DFI 1)*

In another case, a volunteer recognized parallels between her own caring for her family members and the needs of people with dementia at the DFI. This encouraged her to search for ways how people with dementia could maintain a sense of control in a DFI:

*I think it’s important. Because I am a carer for my autistic nephew and my handicapped brother, and for them recognition and being able to have control is so important. So crucial. And here, I see that with people with dementia, too. (volunteer, DFI 1)*

In addition to personal life experiences, participants also discussed professional experiences where emotions such as a sense of personal efficacy played a role, both positive and negative. One volunteer recognized how he applied his former participation management style to his current volunteer work, and how he liked it:

*I always found that to be the fun and exciting part of my job, that you can take people along with you, but that you also … yes, that you notice that employees and students themselves start to participate and help to steer. Yes, that’s delightful. (volunteer, DFI 3)*

Emotions that are connected to dementia itself also played a role. Participants recognized the emotions that come with dementia, such as shame and ‘taboo,’ and how these affected attitudes around involvement:

*They want to hear about it* [the DFI] *and say what they think*. *In their hearts they do, but they just don’t dare. Especially for many carers, it is a taboo. (professional, DFI 2)*

The carers themselves mentioned their personal circumstances per the extent to which they wanted to be involved. Especially, the balance between caring for a relative with dementia and still having time for themselves influenced their perspectives on involvement:

*But I, for myself, wouldn’t like to be busy with all kinds of Alzheimer’s activities every day. I mean, I have a husband with Alzheimer’s and if I … I want to have things for myself that don’t have to do with Alzheimer’s. (carer, DFI 1)*

One carer reflected on her involvement during the development of a DFI and addressed the circumstances behind dropping out:

*It was all too much, and even now I just have to be careful to draw a line, and I really try to draw it. Because … I still have to, there is still so much coming my way. (carer, DFI 2)*

### 3.3 Involvement requires an open, responsive stance and building relationships

Involvement did not happen automatically. All participants were aware that the involvement of people with dementia and/or carers required both verbal and nonverbal communication, such as joining people with dementia during their activities or making contact and observations, listening to conversations during a DFI, asking for specific feedback, inviting a carer as a counselor, and adjusting work routines, if needed:

*Ask them, just ask them, it can be that simple. … You can think of anything, but you can confirm if that’s correct. If you have all kinds of ideas, just ask: ‘Would this be nice for you?’ Or, in case you talk to the carer: ‘Would this be nice for your relative?’ (professional, DFI 1)*

Another professional underlined the importance of listening and being patient until people speak up:

*Just sit among them, ask them, or just listen to what’s being said. And wait. Because things do come up, about the activity or why they’re there. You don’t always have to ask questions about that. You must be invisible. And listen. (project leader, DFI 1)*

Next, involvement also required actions regarding the work context, such as making room in an agenda and adapting work routines. Both a professional and volunteer observed how work routines took priority over the wishes and needs of people with dementia and their carers:

*Agendas, appointments, or I don’t know what. … This morning I spoke to a professional for carers who said, ‘no, I can never go on Wednesdays, because then I always have a meeting.’ I understand that, but the people need to be central, not your agenda. (project leader, DFI 1)*

Similarly, the scheduling of professionals and volunteers in DFIs was based on the interests of the organizations rather than on the preferences of the people with dementia and/or the carers in building relationships:

*R.* [professional] *and L.* [volunteer] *are very well known*; *they were at the DFI from Day 1. And they* [persons with dementia and carers] *asked for them. And then J.* [professional] *said, ‘we don’t want a permanent person at the DFI because that would be difficult; suppose that person drops out.’ (volunteer, DFI 1)*

Verbal and nonverbal communication should be aimed at understanding the person with dementia and their carers for who they are. Such communication requires building relationships and trust:

*It’s not only knowing what people with dementia find interesting but understanding why. (professional, DFI 1)*

*Doing things together, and the connection that arises from it. Dementia-friendly is about building relations. (volunteer, DFI 3)*

The importance of building relationships and trust was underlined in stories from people with dementia and their carers:

*It would have meant so much for me if someone from the club had reached out to me … we could have supported each other, I know that he* [her husband] *can be difficult. But instead, if I arrived at the club, I felt watched and not welcome. It did not give me a nice feeling. (carer, DFI 3)*

Similarly, people with dementia spoke about the ‘room’ they feel to (dare to) take up to express themselves during the DFIs by talking or doing. Feeling trust that you can express yourself was considered important:

*SP 1: And that whole feeling of openness, and being allowed to be who you are, you also have that at the carers’ café*? *SP 3: No, not like that. I think that at the cafe they hover around it, but it’s not clear if they really heard you, understood you. (person with dementia, DFI 2)*

### 3.4 Estimation of one’s own and others’ capacities influences perspectives on involvement

This theme was mentioned only by professionals and volunteers. According to the professionals and volunteers, capacities were needed involving people with dementia and/or their carers. However, there were differences as to whose capacities this concerned. Some professionals and volunteers asked themselves ‘what is required’ to involve people with dementia and their carers, and they reflected on their own capacities to meet those requirements. One professional reflected on her own motivation and capacities:

*If they ask me, ‘Do you want to do that* [create involvement]*?’ Then I’m like, ‘do I want that? And can I? (project leader, DFI 1)*

Another volunteer described what he learned about himself in the attempts to involve people with dementia and their carers:

*Endurance is very important. You have to have a lot of endurance … and I seem to be blessed with that. (volunteer, DFI 3)*

Another professional expressed how reflection and frankness were helpful in creating PPI during the development and sustainment of DFIs:

*Don’t fill in for someone else: that basically sums everything up. And also, frankly confess if you think, ‘oops, I estimated that differently’ or ‘that turned out differently than I expected,’ and start the conversation again. (professional, DFI 1)*

Instead of reflecting on personal capacities, some professionals and volunteers referred to the symptoms of dementia and the decline of cognitive capacities and communication skills. They were concerned about people with dementia being ‘overcharged’ when they were involved in the development or sustainment of DFIs.

One volunteer addressed the need for someone without dementia to interpret the input of the person with dementia during PPI:

*You are dealing with people you know. If you want to make long-term agreements, you will not succeed because their mental capacities are diminishing. And it is always up to the non-demented person to keep an eye on the relationship, and also to consider what one can reasonably expect. And, ‘how do you value his input?’ (volunteer, DFI 3)*

Another professional addressed communication limitations, and that these would be a barrier for PPI during the development and sustainment of DFIs:

*You can always have a say, and people with dementia, also severe dementia, can still indicate what they want or don’t want. Only, in such projects* [developing and sustaining DFIs] *it is sometimes more difficult, isn’t it, because you also have to be able to communicate, you also have to be able to indicate what you want, and if you have a serious form of dementia. … So you do need that someone who can communicate. (professional, DFI 2)*

Most participants regarded the carer as a valuable advisor or substitute for the person with dementia:

*I think that informal carers know their partner or their loved one with dementia better, so that they can give their feedback much better and think along with them, yes, people with dementia … because they are usually, yes, quite far gone. (volunteer, DFI 1)*

However, some participants doubted whether carers also had the capacity to participate in the development of DFIs:

*But I also know carers who might want to, but simply don’t have the competence to really participate. (volunteer, DFI 2)*

## 4 Discussion

The purpose of this study was to increase our understanding of the perspectives of all stakeholders, including people with dementia and their carers, on the involvement of people with dementia and their carers in the development and sustainment of community DFIs (dementia-friendly initiatives). We found four themes that reflect their perspectives, namely 1) the involvement of people with dementia and their carers is important for both people with dementia and their carers and other stakeholders; 2) personal character traits, life histories and associated emotions evoke the need for involvement; 3) involvement requires an open, responsive stance and building relationships and 4) the estimation of one’s own and others’ capacities influences perspectives on involvement.

A cross-cutting thread in these themes is relational expertise and relational agency, as conceptualized by Anne Edwards (52). Relational expertise is the ability to recognize the expertise of specific groups and form connections with them, enabling the leveraging of their expertise (52). Relational agency refers to the ability to understand the meaning of others’ concerns, actively listen to their interpretations of a situation, and increase their ability to respond to them (52). These concepts align with our results, as the involvement of people with dementia and their carers was important for understanding their needs. It fostered reciprocity and reward between people with dementia and carers and other stakeholders. As such, it increased connections and the ability to understand the concerns and needs of people with dementia and their carers. Next, personal character traits, life histories, and associated emotions that evoke the necessity for involvement were intertwined with the ability to understand the meaning of others’ concerns and the leveraging of their expertise through the recognition of personal values and preferences. Furthermore, the involvement required an open and responsive stance, emphasizing the building of strong relationships. Such an approach fostered trust, communication, and collaboration among all stakeholders, creating an environment conducive to shared decision-making and mutual support. Finally, the estimation of one’s own and others’ capacities played a pivotal role in recognizing and valuing expertise and agency within relationships and collaboration. Required was the ability to both listen to others and enable them to respond and share expertise.

Relational expertise and - agency foster reflection and self-awareness that deepens understanding of interpersonal dynamics (53). Through interpersonal connections and self-reflection, people can build a stronger foundation for positive self-perception and the ability to navigate social interactions with increased confidence and authenticity (22, 23, 53, 54). In our study, participants reflected on interpersonal dynamics during DFIs and how these affected their confidence or authenticity. An example of confidence was portrayed when a professional talked about restarting the conversation after acknowledging that things turned out differently than expected. An example of authenticity is the lack of it in the quote where a volunteer could not work ‘from the heart’, as (s)he wanted to, due to diminished spontaneity. The importance of working relationally resonates with the literature about PPI in the field of dementia-friendliness (13, 14, 18–20, 55).

In our study, participants shared different opinions on the extent that intellectual and cognitive capacities of people with dementia and their carers play a role in being involved.

On the one hand intellectual and cognitive limitations were seen as a barrier to keep up the usual practice of the stakeholders; conversely, emotional and cognitive abilities were acknowledged by stakeholders as ‘input’ for acclimatization and communication with people with dementia and their carers. In fact, in the first case, people with dementia and their carers were stereotyped and considered unable to fit in the practices of stakeholders; in the second case, stakeholders were committed to respond to the capacities of people with dementia. This points to how some stakeholders might be very well informed about the needs of people with dementia and carers based on their limitations, but may overlook the lived experience. Without the focus on lived experience, the interaction between stakeholders and people with dementia and carers can become, rather, an ‘impersonal service-need relationship’ (56). Those relations do not reflect the real purpose of dementia-friendliness in which inclusion, integration and equity of people with dementia and carers is central (8, 18, 54, 57). Instead, this purpose was reflected in the relationships and communication of other stakeholders who truly believed that people with dementia and their carers can make meaningful contributions, like any other person or stakeholder. Characteristic in these relations and communication was that success depends on social, cognitive and emotional skills rather than intellectual efforts (53, 56, 58). Involving people with dementia and their carers during the development and sustainment of DFIs is a way to build relationships and, feel included and valued as citizens (8, 23, 59). A personalized customized relationship during involvement between stakeholders and people with dementia and carers makes a real difference in becoming dementia-friendly.

The need to structure PPI has been emphasized to improve PPI during building DFIs and DFCs (12, 15, 18–20) Instead of a focus on working relationally, frameworks or models of stakeholder management should underpin how the involvement of people with dementia and carers should take place. Indeed, an organizational approach could help to organize PPI during the development of a DFI. However, it is crucial that PPI is grounded in a rights-based agenda perspective and is integrated with the beliefs of people in the organization, in order to avoid simply ‘ticking boxes’ (8, 56, 60). A lack of this was visible in the quote of a volunteer who disliked the amount of feedback for not being able to act spontaneously. In another quote, some professionals were using organizational circumstances such as agendas or staff scheduling as an excuse when people with dementia asked about the presence of two valued helpers. In that sense that quote illustrates the hope of people with dementia to build or strengthen relationships. It also shows that adding organizational structure PPI alone is not sufficient to understand how and why people with dementia want to affect the DFI. This underlines the need for a culture change to support PPI for people with dementia and carers. The cultural and organizational change should especially move away from the legacy and structure of service-led working cultures towards more user-led organizations that develop routines which allow people with dementia and carers to be themselves, and where the relationship is a genuine partnership (13, 18, 20, 22, 60–62). Such focus could be enriched by theories from situated learning, (e.g., workplace learning (66) or social theories, such as social health, assets- based community development or social capital (31, 63). These theories recognize the importance of personal growth, team dynamics, and societal contributions. By integrating these elements in developing and sustaining DFIs, stakeholders and people with dementia and their carers can create meaningful change that benefits themselves, other stakeholders, and the broader community. This requires context sensitivity, accessible places to meet, and creativity in methods of involvement that support working relationally (15, 68, 69). It is also an opportunity for a positive approach in developing and sustaining DFIs, for example by giving positive feedback to people and places or by exchanging positive experiences and resources (10, 19). Such positive belief (e.g., in an assets- based approach with a focus on the impact on people) reflects the purpose of dementia-friendliness and enables the sustainment of PPI during DFIs by shared learning, responsiveness, and the tailoring of activities (18, 19, 62).

### 4.1 Methodological strengths and limitations

This is one of the first studies to look at PPI in DFIs in which both perspectives of stakeholders and people with dementia and their carers are central. We considered personal factors affecting involvement including the why and how of involvement. For this, the co-research design and the role of the caregiver as co-researcher was a strength. We collaborated with the co-researcher as a lay expert during a shared decision-making process. Instead of from a trained researchers’ perspective or consultation model, the co-researcher reflected and commented from a lay, or outsider perspective, on the research process and -results (26, 33, 64). As such, he stimulated and offered analytic and interpretative insights which may otherwise not have been visible for the professional researchers. We could have improved our process by adding more co-researchers for a better balance of perspectives. Possibly, a co-researcher with an interest in research could have performed research tasks such as interviewing and coding.

We achieved a good range in terms of different stakeholders’ roles (e.g., professional, volunteer, carer, and people with dementia and the distribution per DFI) which was important in reaching saturation (65–67).

We could have included a more diverse range of stakeholders with different cultural backgrounds and, also, non-participants of DFIs who could have shared valuable views and insights on involvement. Diversity enriches the data by introducing a wider range of perspectives, experiences, and cultural insights. This could have enhanced the depth of the analysis, thereby achieving better saturation (65–67).

Also, our sample was limited to three Dutch DFIs in which two were dementia-specific and one was dementia-inclusive. Due to time constraints, we were unable to recruit equal numbers of both dementia-specific or - inclusive initiatives. Conversely, the predominance of dementia-specific DFIs does fit with Dutch practice where dementia-specific initiatives seem to be common (68).

We did not recruit DFIs on the implementation of PPI, for example, based on who should have been involved, or how people with dementia and carers should have been involved. Most participants did not have extensive experience with implementation of PPI, which might influence their perspective on the PPI of people with dementia and carers. Insights into the implementation of PPI might have enabled a deeper understanding of PPI by people with dementia and carers.

Lastly, this study’s people with dementia and carers were also (former) users of the DFIs. As such, their input was also influenced by their participation in DFIs, and might have been biased by their positive or negative experiences within those (18).

### 4.2 Practical implications

Our study underscores the need for working relationally for the PPI of people with dementia and their carers in DFIs. What is needed are dedicated DFIs aimed at solidifying involvement as part of increasing dementia-friendliness in the community. The development and sustainment of these DFIs should be characterized by a more user-led organization and a genuine partnership. For people with dementia and carers, such an organization enables a safe environment with a shared group identity around a collective perspective, narrative, or goal (18, 20, 23). It is crucial that people with dementia and carers understand how their involvement will make a difference. This may also prevent withdrawal (15) and/or encourage new people to get involved (18, 61) - as there will be a turnover of people with dementia being involved for as long as they are willing or able - and create more diversity among the people with dementia and carers.

For other stakeholders, offering a range of different ways to facilitate involvement might enable a wider group of people with dementia to get involved (18, 69) for example, through their cultural background or via the severity of dementia. For this, resources such as creative methods, problem solving, and communication skills are important (19, 60, 70, 71).

### 4.3 Further research

Our study suggests that stakeholders’ and people with dementia and carers’ beliefs, emotions, and reflections play a significant role regarding the involvement of people with dementia and their carers in DFIs. Future researchers might conduct longitudinal studies to evaluate how involvement, relational dynamics, and outcomes evolve over extended periods. This would help in understanding the long-term impact of DFIs on the well-being of people with dementia and their carers.

Future studies might also investigate the implementation of PPI in depth and delve into the strategies, challenges, successes, and failures of implementing PPI in various dementia-friendly initiatives. Additionally, such a study could aim to include a greater diversity of stakeholders and non-participants of DFIs to provide a more comprehensive understanding of how involvement practices contribute to the overall outcomes of DFIs. Finally, enhancinge the co-research design by increasing the number of co-researchers with diverse backgrounds might achieve a better balance whole team balance of perspectives and research skills.

Pursuing these directions in future research would contribute to a deeper understanding of perspectives on the involvement of stakeholders, people with dementia, and carers in community DFIs. The inclusion of people with dementia and their carers would increase, too.

## 5 Conclusion

This study examined the perspectives of stakeholders, people with dementia, and carers on the involvement of people with dementia and their carers in community DFIs. An important conclusion can be drawn from the study; the involvement of people with dementia and carers requires working relationally in the development and sustainment of DFIs;

Overall, the results of this study can support practices in analyzing or reflecting on their current or future practices when developing or sustaining DFIs. Additionally, our results might help stakeholders both monitor the process of involvement and inspire them to take a positive or assets-based approach.

## Data availability statement

The raw data supporting the conclusions of this article will be made available by the authors, without undue reservation.

## Ethics statement

The study was approved by the research ethics committee of the Radboud University Nijmegen Medical Centre desicion number: 2021-13349. The studies were conducted in accordance with the local legislation and institutional requirements. Written informed consent for participation in this study was provided by the participants’ legal guardians/next of kin.

## Author contributions

MT: Conceptualization, Data curation, Formal analysis, Funding acquisition, Investigation, Methodology, Project administration, Resources, Software, Validation, Visualization, Writing – original draft, Writing – review & editing. LD: Conceptualization, Formal analysis, Methodology, Writing – review & editing. FL: Investigation, Methodology, Resources, Writing – review & editing. MN-VS: Resources, Supervision, Writing – review & editing. RD: Funding acquisition, Resources, Supervision, Writing – review & editing. MG: Resources, Supervision, Writing – review & editing. WK-S: Conceptualization, Resources, Supervision, Writing – review & editing.
