# Supplementary material for: ‘Practice what you preach’. Perspectives on the involvement of people with dementia and carers in community-based dementia friendly initiatives, a qualitative study
Source: Front Psychiatry. 2024 May 16;15:1387536. doi: 10.3389/fpsyt.2024.1387536 (PMC11137317; doi:10.3389/fpsyt.2024.1387536)
Supplement: Supplementary file 1 [file Presentation_1.pdf]

Supplementary Material: Interview guide about the involvement of people with dementia and/or their informal caregivers in the development or sustainment of dementia-friendly initiatives

'Practice what you preach'. Perspectives on the involvement of people with dementia and carers in community based dementia friendly initiatives, a qualitative study.

Authors: Marjolein Thijssen* [1,2,3], Linda Dauwerse[4], Frans Lemmers[5], Ria Nijhuis-van der Sanden[1]†, Ramon Daniels[6] †, Maud Graff [1,2]†, Wietske Kuijer-Siebelink [7,8].

Interview guide about the involvement of people with dementia and/or their informal caregivers in the development or sustainment of dementia-friendly initiatives

Belonging to Mentality; a study into success factors of dementia-friendly projects and communities.

Instruction:

Use the concretization and examples below in the conversations.

**Propper (2009)**	**Rol**	**Hoe**
Participate[1]	Collaboration partner	What: Participate in decision-making and implementation.<br><br>Via: organizing, implementing and evaluating plans and activities.<br><br>Core: Joint decisions
Participate in decision-making	Co-decision maker	What: Assessing facts, visions, goals, solutions and choices and being able to help make choices.<br><br>Via: meetings, evaluations, (project) group meetings<br><br>Core: Assessment plays a role in decision-making
Think along[2]	Initial advisor (feed forward)	What: Being asked about needs, being allowed to formulate a problem, giving advice at the start of the process (initial consultation)<br><br>Via advisory committee, citizens' panel, conference.<br><br>Core: Advice provides guidance but is not binding for decisions.
Co-assess	Desicion advisor feedback	What, Being consulted, being allowed to give an opinion afterwards (final speech)<br><br>Via: interviews, survey, discussions, polls ect.<br><br>Core: Opinions are noted, but are not binding on decisions.
Knowing	Spectator, receiver of information	What: Having access to information<br><br>Via: e.g. newspapers, internet, visits, leaflets, brochures, etc<br><br>Core: Do not provide input

[1] Propper puts participation at the highest level, while Arnstein sees co-decision making as the highest level. However, participating involves more equality and continuity.
[2] Think along is more important than co-assess; co-assessment is mainly about opinions about the results afterwards. The importance of thinking along is also more decisive for follow-up.

Because of the semi structured nature of this interview guide and the diversity in background of participants, each interview may have its own course. However, the questions that should be asked at a minimum are in bold.

Regarding interview techniques:

- Listen carefully and choose the terms that best suit the participant. For example: if the term ‘initiative’ is not clear enough, opt for ‘activity’ or the word the participants are using themselves. This will make the conversation easier.

- Summarizing is a very important interview technique, because you give the participant a recap of what you have heard. But you have to bear in mind that the objective is not to come up with a comprehensive summary. For that reason, make sure your summary is concise and to the point.

Introduction

Thank you for taking the time to do this interview today. As you probably know, many municipalities are working to ensure that people with dementia and informal caregivers remain an active part of their neighborhood and continue to belong. To this end, dementia-friendly initiatives and activities are developed that are a kind of 'building blocks' in building a dementia-friendly district/neighborhood. You are involved in one of those initiatives, namely [name dementia-friendly initiative]. That initiative is central to our conversation.

The interview is about the involvement of people with dementia and/or their informal caregivers in the development of dementia-friendly initiatives, in other words the extent and way in which they have a say.

We would very much like to hear your possible experiences, opinions and needs regarding the involvement and efforts of people with dementia and their informal caregivers during [name dementia-friendly initiative]. It is not a conversation where answers are right or wrong, we want to learn from you and all your answers are very valuable to us.

Start

Key question: Do you think it is important that there are dementia-friendly initiatives and that the target group is involved?

Questions Professionals/Volunteers	Questions people with dementia and their carers

Do you think it is important that there are such initiatives?<br><br>Can you explain that?<br><br><br>Do you think it is important that people with dementia and/or their informal caregivers are involved in the development of dementia-friendly initiatives? Or have a say in some way?	Do you think it is important that there are such initiatives, such as [name dementia-friendly initiative].?<br><br>Can you explain that?<br><br>If not: why not, what does it evoke in you? what are your needs?<br><br><br>Do you think it is important that you can have a say in such activities? Or: can you indicate your needs and participate in the discussion?

Explanation of involvement/commitment/participation for transparency and clarity
Involvement and commitment of people with dementia can be broad. For the sake of clarity, I will explain:<br><br>People with dementia and informal caregivers **can be informed** and are then **recipients of information**. They can also be involved as an **advisor or assessor** of plans and ideas, or as a **co-decision maker** or **participate as a collaborative partner** in decision-making and implementation.

Part 1: Past; Experiences & (former) knowledge (15 minutes)

Key question: Do you have previous experiences with having a say in similar activities, for example from work, volunteer work or otherwise?

Questions professionals/volunteers	Questions people with dementia and their carers
Have you been involved in similar initiatives before? For example: organized? or advice given? Or asked for your opinion, possibly with a questionnaire? How was that? How did you experience that? Did that also play a role in your decision to participate again? How did your previous experience influence this participation?<br>Why did you participate? What was the reason/motivation/important for doing that?<br><br>Possibly also in other activities, e.g. from your work, volunteer work or otherwise?<br>If so, what was that? How did it go? How did you find that to do?<br>How was that? How did you experience that? Did that also play a role in your decision to participate again? How did your previous experience influence this participation?	Have you had a say in activities that took place in your municipality? For example: Have you ever helped organize an activity? or advice given? Or have you ever been asked what you thought of the activity, possibly with a questionnaire? or have you been asked what you find important? Why did you participate? What was the reason/motivation/important for doing that?<br><br>Have you ever been to the working group that organized the initiatives/activities?<br>What was it like to be in such a group? OR<br>Would you have liked to have been there?<br>If so<br>How do you feel that you have had a say? Do you think it is important that you had/were given the opportunity to do so? That you were asked to do this? Can you explain that?<br>If not<br>How do you feel about not having had a chance to do that? you're not asked for? Do you think you should have been involved in your participation?<br>Have you been involved in initiatives before? Have you had a say in activities before? For example: organized? or advice given? Or asked for your opinion, possibly with a questionnaire? E.g. from your work, volunteer work or otherwise?<br>If so, what was that? How did it go? How did you find that to be done?<br>If not: Would you have liked that? What would you have done then?

Part 2: Present: Values, beliefs, attitude, personal perception (30 min)

Key question: how are you involved in this initiative and how important do you think it is to have a say? What do you believe in, in terms of participation?

Questions professionals/volunteers	Questions people with dementia and their carers
Since when have you been involved? How did you get involved? How did you become involved?<br>What was your reason/motivation to participate? Why did you participate?<br><br>What is/has been your contribution/role/input in the development/sustainability of the activity? What do you do in development/sustainability? What is done with that? How will this be followed up?<br><br>Has the involvement and commitment of people with dementia or informal caregivers been discussed or discussed? In any form? When? (How) did/not that get a follow-up?<br>Why did it turn out this way, in your opinion? Who or what influenced/was decisive?<br><br>What do you think of the (possible) participation/involvement of people with dementia and/or informal caregivers? What is your position regarding the (possible) involvement and efforts of people with dementia and/or informal caregivers? Can you tell us more about that?<br>Are there certain beliefs, insights or experiences underlying it? Do they play an	Do you think it is important to have a say/be involved in these activities? Tell me? Why is that important to you?<br><br>Are you currently involved in the activity? Do you have a say in the activity? How is that organized/arranged? What are you talking about?<br>What will be done with your input for the activity? How is your input used in development/sustainability?<br><br>Would you like to have a say in the activity at this time? How is that important for you?<br>If so: How would you like to have a say/be involved? For example: help organize/advise/give your opinion?<br>About what/when/with whom?<br><br>What should they do with your input?<br>If not:<br>Tell me? Do you not want to be involved/have a say? Could others do it in a different way? Or Is there someone else who speaks for you?/represents you? What will he/she mainly pass on?<br>Or do you not want to be involved in these activities anyway? Can you explain that?

(important) role in this? Can you give an example?<br><br>What do you think of the efforts, also at policy level, to involve the target group as much as possible?<br><br>Are there certain roles that you think are more/less obvious for people with dementia and their informal caregiver? Is there certain involvement/participation that you do not consider feasible? What is important? plays a role in this?<br><br>Is there a distinction between a person with dementia and an informal caregiver? What do you think is the reason for this? Why do you think that?	What do you think of the efforts, also at policy level, to involve the target group as much as possible?
Would it have made a difference to you personally if people with dementia and/or informal caregivers had been involved? Can you explain that?<br><br>OR:<br><br>What has the involvement and commitment of people with dementia and/or informal caregivers brought you personally?	Would it make a difference to you personally if you were involved/had a say? Can you explain that?<br><br>OR<br><br>What has it brought you personally to be (or has been?) involved?

Part 3: Future: Needs and dreams

Key question: what should the ideal world look like for people with dementia and informal caregivers?

Questions professionals/volunteers	Questions people with dementia and their carers
Future: what should the future look like for people with dementia and informal caregivers? How could people with dementia/informal caregivers be involved/have a say in this? Do you have an example of that? How do you envision that?<br><br>How should that be addressed? What could your role be in this? How could you contribute to this yourself?<br><br>What would you need, what would be helpful?<br><br>What lessons can we learn from the past and present?	Future: what should the future look like for people with dementia and informal caregivers? How could people with dementia/informal caregivers be involved/have a say in this?<br><br>How should that be addressed? What could your role be in this? How could you contribute to this yourself?<br><br>What would you need, what would be helpful?

Review of conversation:

Key question: Were you able to answer questions, did you miss any questions? Would you like to emphasize or add anything in your answers?

Finally: some questions about yourself: (record answers as well, note that you don't have to ask what you already know, you can repeat it)

Questions professionals/volunteers	Questions people with dementia and their carers
May I ask to know your age? …years<br><br>You are Male Female<br>I'd rather not say<br><br>How would you describe your cultural background?<br><br>Dutch, European Asian African<br>American Otherwise, namely…….<br><br>What is your highest education?<br><br>University<br>Higher professional education<br>Vocational education<br>Lower vocational education<br>Otherwise, namely…………	May I ask to know your age? …years<br><br>You are Male Female<br>I'd rather not say<br><br>How would you describe your cultural background?<br><br>Dutch, European Asian African<br>American Otherwise, namely…….<br><br>What is your highest education?<br><br>University<br>Higher professional education<br>Vocational education<br>Lower vocational education<br>Otherwise, namely…………
