# Supplementary material for: ‘Practice what you preach’. Perspectives on the involvement of people with dementia and carers in community-based dementia friendly initiatives, a qualitative study
Source: Front Psychiatry. 2024 May 16;15:1387536. doi: 10.3389/fpsyt.2024.1387536 (PMC11137317; doi:10.3389/fpsyt.2024.1387536)
Supplement: Supplementary file 2 [file Presentation_2.pdf]

Supplementary Material: Overview of themes and subthemes

‘Practice what you preach’. Perspectives on the involvement of people with dementia and carers in community based dementia friendly initiatives, a qualitative study.

Authors: Marjolein Thijssen* [1,2,3], Linda Dauwerse[4], Frans Lemmers[5], Ria Nijhuis-van der Sanden[1]†, Ramon Daniels[6] †, Maud Graff [1,2]†, Wietske Kuijer-Siebelink [7,8].

	**Themes**	**Subthemes**
1	the involvement of people with dementia and their carers is important for both people with dementia and their carers and other stakeholders	Involvement of the target group in DFI is important so that the target group has a good time and feels heard and seen.
2	personal character traits, life histories and associated emotions evoke the need for involvement	Personal character traits of the person
		Personal experiences with emotional impact influence attitudes towards involvement and participation
		Personal experiences of impact regarding enabling involvement or having a say.
3	involvement requires an open, responsive stance and building relationships	Ask and listen, sit with pwd to increase involvement and participation. Questioning carers of people with dementia.
		That you make room in your agenda and working method for people with dementia and ther carers
		Space that is felt by people with dementia to be able to say what you think and feel and/or to act according to how you feel.
		Emotions regarding dementia influences expressing needs/opinions
		Personal choices regarding activities influence the extent to which carer wants to be involved
4	the estimation of one’s own and others’ capacities influences perspectives on involvement.	Reflection on own capacities and capabilities by professionals and volunteers
		Estimation of capacities of people with dementia and carers
